# Supplementary material for: Comparative multiomics analysis of cell physiological state after culture in a basket bioreactor
Source: Sci Rep. 2022 Nov 23;12:20161. doi: 10.1038/s41598-022-24687-4 (PMC9686226; doi:10.1038/s41598-022-24687-4)

23666053.1:33242578:33242676:-@NW_023666053.1:33242578:33242694:-@NW_023666053.1:33243421:3324[illegible]

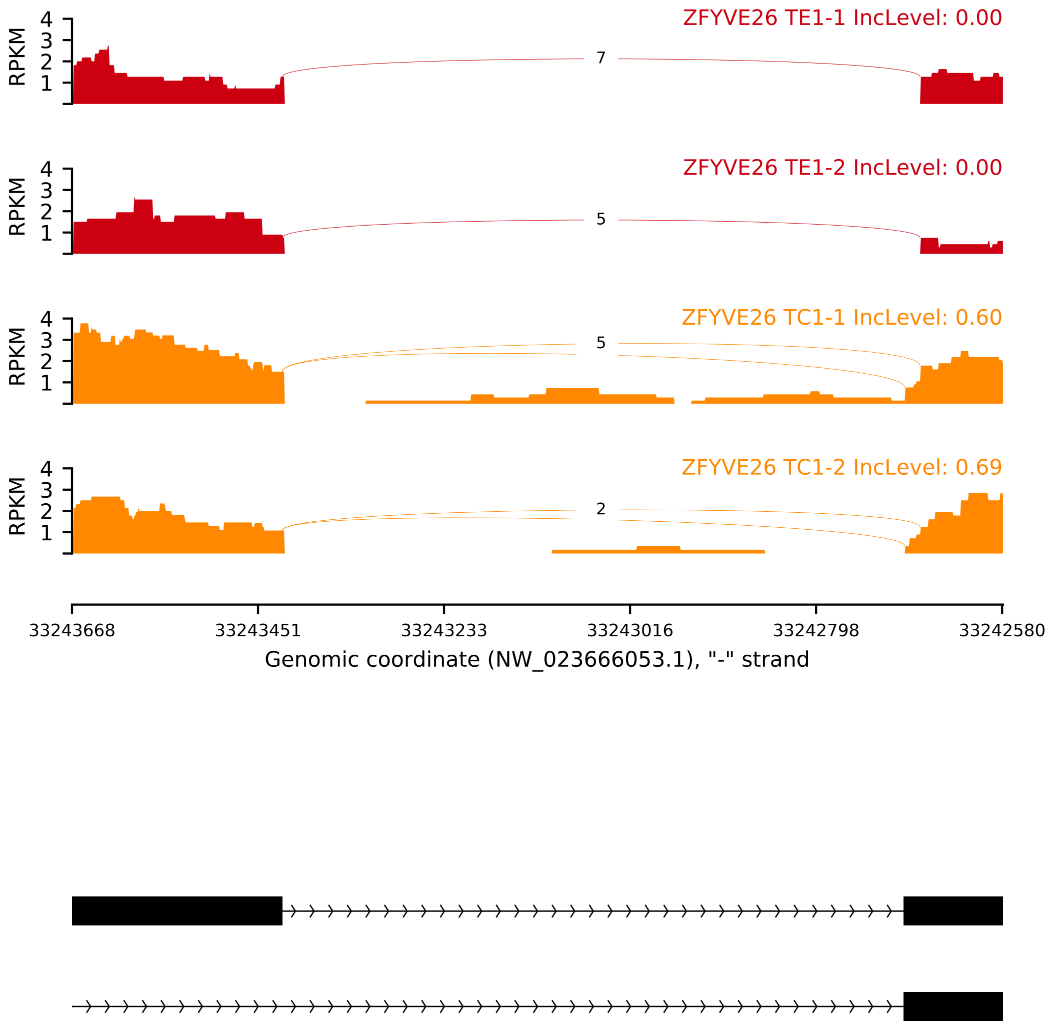

Supplement: Supplementary file 1 — Supplementary Information 1. [file 41598_2022_24687_MOESM1_ESM.zip › raw data/Transcriptomic raw data/7.AS/2.dasplot/TE1vsTC1/A3SS/11_ZFYVE26_NW_023666053.1_33242578_33242676_-@NW_023666053.1_33242578_33242694_-@NW_023666053.1_33243421_33243667_-.pdf]
